# TMPRSS2 Expression and Activity Modulation by Sex-Related Hormones in Lung Calu-3 Cells: Impact on Gender-Specific SARS-CoV-2 Infection

**DOI:** 10.3389/fendo.2022.862789

**Published:** 2022-05-31

**Authors:** Donatella Treppiedi, Giusy Marra, Genesio Di Muro, Rosa Catalano, Federica Mangili, Emanuela Esposito, Anna Maria Barbieri, Maura Arosio, Giovanna Mantovani, Erika Peverelli

**Affiliations:** ^1^ Department of Clinical Sciences and Community Health, University of Milan, Milan, Italy; ^2^ PhD Program in Endocrinological Sciences, University Sapienza of Rome, Rome, Italy; ^3^ PhD Program in Experimental Medicine, University of Milan, Milan, Italy; ^4^ Endocrinology Unit, Fondazione Istituto di Ricovero e Cura a Carattere Scientifico (IRCCS) Ca’ Granda Ospedale Maggiore Policlinico, Milan, Italy

**Keywords:** SARS-CoV-2, Spike, TMPRSS2, androgen, estradiol

## Abstract

Coronavirus disease 2019 (COVID-19) is caused by severe acute respiratory syndrome coronavirus 2 (SARS-CoV-2). Although males and females are at equivalent risk of infection, males are more prone to develop a higher severity disease, regardless of age. The factors that mediate susceptibility to SARS-CoV-2 and transmission are still under investigation. A potential role has been attributed to differences in the immune systems response to viral antigens between males and females as well as to different regulatory actions played by sex-related hormones on the two crucial molecular effectors for SARS-CoV-2 infection, TMPRSS2 and ACE2. While few and controversial data about TMPRSS2 transcript regulation in lung cells are emerging, no data on protein expression and activity of TMPRSS2 have been reported. Aim of the present study was to search for possible modulatory actions played by sex-related hormones on TMPRSS2 and ACE2 expression in Calu-3 cells, to test the effects of sex-steroids on the expression of the 32kDa C-term fragment derived from autocatalitic cleavage of TMPRSS2 and its impact on priming of transiently transfected spike protein. Cells were stimulated with different concentrations of methyltrienolone (R1881) or estradiol for 30 h. No difference in mRNA and protein expression levels of full length TMPRSS2 was observed. However, the 32 kDa cleaved serine protease domain was increased after 100 nM R1881 (+2.36 ± 1.13 fold-increase vs control untreated cells, p < 0.05) and 10 nM estradiol (+1.90 ± 0.64, fold-increase vs control untreated cells, p < 0.05) treatment. Both R1881 and estradiol significantly increased the activating proteolytic cleavage of SARS-CoV-2 Spike (S) transfected in Calu-3 cells (+1.76 ± 0.18 and +1.99±,0.76 increase in S cleavage products at R1881 100nM and 10 nM estradiol treatment, respectively, p < 0.001 and p < 0.05 vs control untreated cells, respectively). Finally, no significant differences in ACE2 expression were observed between hormones-stimulated cells and untreated control cells. Altogether, these data suggest that both male and female sex-related hormones are able to induce a proteolityc activation of TMPRSS2, thus promoting viral infection, in agreement with the observation that males and females are equally infected by SARS-CoV-2.

## Introduction

Coronavirus disease 2019 (COVID-19) is caused by the novel severe acute respiratory syndrome coronavirus 2 (SARS-CoV-2) (1). Even though the majority of people infected with SARS-CoV-2 are asymptomatic or present with mild symptoms and do not require hospitalization, in a subset of patients the clinical features may progress to acute respiratory distress syndrome (ARDS) and cardiac injury. Since the beginning of the pandemic, COVID19 has caused hundreds of thousands of deaths worldwide. Epidemiological studies have identified major risk factors for developing severe symptoms such as age, obesity, diabetes, hypertension, respiratory or cardiovascular diseases and sex (2–6). Although the percentage of confirmed cases has been reported to be equal among men and women, for every 14 males confirmed cases that have died from COVID-19 there are only 10 female (https://globalhealth5050.org/the-sex-gender-and-covid-19-project/the-data-tracker/) (Global Health 5050, 2021). This evidence has suggested the importance of considering sex gender as a critical variable in the clinical research to defeat SARS-CoV-2 pandemic. There are at least two possible mechanisms by which androgens may determine clinical outcomes in COVID-19. The first plausible mechanism is related to androgen-driven immune modulation. The second possibility refers to differences between males and females in the expression levels and genetic variants in angiotensin-converting enzyme 2 (ACE2) receptor and cellular serine protease TMPRSS2, the two crucial genes for viral infection (7, 8).

Indeed, as other SARS-CoV, SARS-CoV-2 cells entry and infection rely on recognition and attachment of the viral spike (S) glycoprotein to the ACE2 transmembrane protein on host cells and engagement of the TMPRSS2 protease for S protein priming (8–13). Briefly, entry depends on binding of the surface unit, S1, of the S protein to ACE2 receptor, that in turn promotes viral attachment to the plasma membrane of target cells. In addition, S protein priming by cellular proteases is required. The S protein cleavage at the S1/S2 and the S2’ sites allows fusion of viral and cellular membranes, a process specifically driven by the S2 subunit (8). TMPRSS2 is made as a precursor protein (zymogen) of 70 kDa which undergoes autoproteolytic activation releasing the 32 kDa fragment containing the protease domain into the extracellular space (14, 15).

Both ACE2 and TMPRSS2 have been suggested to be implicated in the modulation of susceptibility to SARS-CoV (16, 17) and SARS-CoV-2 as well (8). These genes are predicted to mediate sex-related effects: ACE2 is located on the X chromosome, whilst TMPRSS2 gene has a 15 bp androgen response element (ARE) and is also regulated by estrogen stimulation (18–20). TMPRSS2 is largely expressed in epithelial cells of the respiratory, gastrointestinal, and urogenital tract (21, 22). In prostate cancer cells, TMPRSS2 is strongly upregulated in response to androgens (14, 19, 23). Although no changes in the TMPRSS2 transcript was observed in lung cell lines and lung from mice upon androgen stimulation and inhibition of androgen receptor (24, 25), the expression of TMPRSS2 in the lung was found slightly increased in males (7) and significantly upregulated by androgens in A549 lung cells (26). However, so far, no data about the impact of sex-related hormones on the status of activation of TMPRSS2 are available in lung cells.

In the preset study we investigated the effects of sex-related hormones on ACE2 and TMPRSS2 expression and proteolytic activation.

## Materials & Methods

### Cell Culture and Cell Transfection

The non-small-cell lung cancer cell line Calu-3 (ATCC HTB-55) was kept in culture with Eagle’s Minimum Essential Medium (EMEM, ATCC, Carlsbad, CA) supplemented with 10% fetal bovine serum (FBS) and pen/strep (Life Technologies, Carlsbad, CA, USA) at 37°C and 5% CO_2_ in a humidified atmosphere.

For experiments, cells were seeded in lipophilic hormones-free complete medium consisting of Eagle’s Minimum Essential Medium supplemented with 2% charcoal-stripped bovine serum (CSS) (Merck, Darmstadt, Germany) and antibiotics. Estradiol (17β-estradiol) and R1881, also known as methyltrienolone, were purchased from Abmole Bioscience (Houston, TX, USA) and used as estrogen receptor and androgen receptor agonists, respectively. Expression vector coding for wild-type SARS-CoV-2 Spike (SARS-CoV-2 S) was kindly provided by Prof. Elisa Vicenzi (IRCCS San Raffaele Hospital, Milan, Italy) and transiently transfected in Calu-3 cells using Lipofectamine 2000 (Invitrogen, Thermo Fisher Scientific, Waltham, MA, USA) as transfection reagent, according to the manufacturer’s instructions.

### Quantitative Real-Time RT-PCR

The RNeasy Plus Mini Kit (Qiagen, Hilden, Germany) was used to extract total RNA from Calu-3 cells. Total RNA concentration and purity were measured using NanoDrop Lite Spectrophotometer (Thermo Fisher Scientific, Whaltam, MA, USA). RNA integrity was assessed by 1% agarose gel electrophoresis. Total RNA (1 μg) was reverse transcribed with RevertAid H Minus First Strand cDNA Synthesis Kit (Thermo Fisher Scientific, Whaltam, MA, USA). qRT-PCR was carried out using the SsoFast™ EvaGreen^®^ Supermix (Bio-Rad Laboratories, Hercules, CA, USA) following the instructions of the manufacturer, in a CFX Connect™ Real-Time PCR Detection System (Bio-Rad Laboratories, Hercules, CA, USA). Specific primers were designed for human TMPRSS2, aromatases (CY19A1), androgen receptor and estrogens receptors. All reactions were performed in triplicate and media of CT values was determined. GAPDH was used as housekeeping gene for the normalization of target genes using the Bio-Rad CFX Manager software.

### Western Blot Analysis of TMPRSS2, ACE2 and Spike

Cells were seeded in 6-well plates at a cell density of 2×10^5^/well in EMEM supplemented with 2% CSS and antibiotics. Cells were maintained in this lipophilic hormones-free medium for 7 days, with two medium changes, at day 4 and at day 7, before the start of treatments. For transfection experiments, cells were transfected with plasmid encoding SARS-CoV-2 S at day 5 and left incorporating plasmidic DNA for 2 days before the start of treatments. For each experiment, cells were treated at day 7 with estradiol (10 nM and 100 nM) or R1881 (10 nM and 100 nM) for 30 h in CSS medium. Fresh stimuli were re-added at 24 h of treatments. Doses of estradiol and R1881 as well as time of stimulation were chosen on the bases of preliminary time/concentration dependent experiments (**Supplementary Figure 1**) and literature data (14, 23). Cells were then lysed with lysis buffer. After quantification by BCA assay, 30 µg of total proteins extracted were separated on SDS/polyacrylamide gels and transferred to a nitrocellulose filter. TMPRSS2 antibody was from Santa Cruz Biotechnology (Dallas, TX, USA) and diluted at 1:200, Spike antibody was from GeneTex (Irvine, CA, USA) and used at 1:1000 dilution, ACE2 antibody was from R&D Systems (Minneapolis, MN, USA) and used at 1:1000. The incubation of primary antibodies was carried out at 4°C for 18 h, whilst secondary antibodies anti-mouse or anti-rabbit (Cell Signaling Technology, Danvers, MA, USA) were used at 1:2000 and incubated at room temperature for 1 h. The anti-GAPDH antibody (Ambion, Thermo Fisher Scientific, Waltham, MA, USA) used for normalization was diluted 1:4000 and incubated for 1 h at room temperature. ChemiDOC-IT Imaging System (UVP, Upland, CA, USA) was used to detect chemiluminescence and NIH ImageJ software to analyse the intensity of the bands.

### Immunofluorescence

Calu-3 cells were seeded in 6-well plates at the density of 2x10^5^ cells/well in EMEM supplemented with 2% CSS and antibiotics. Cells were maintained in this lipophilic hormones-free medium for 7 days, with two medium changes at day 4 and at day 7, before start of treatments. For the experiments, cells were treated at day 7 with estradiol (10nM) or R1881 (100nM) for 30 h in CSS medium. Fresh stimuli were re-added after 24 h. After the treatment, cells were trypsinized, counted and re-seeded on 13-mm poly-L-lysine coated coverslips at the density of 1.25 × 10^5^ cells/well in 24-well plates and grown at 37°C for 18 h. The following day, cells were fixed with 4% paraformaldehyde (Sigma-Aldrich, St. Louis, MO, USA) for 10 min at room temperature, washed three times with PBS, and incubated for 1 h at room temperature with blocking buffer (5% FBS, 0.3% Triton™X-100, in PBS).

For immunofluorescence analysis of TMPRSS2 and ACE2 in Calu-3 cells, rabbit anti-TMPRSS2 (1:100, Proteintech, Germany GMBH) and rabbit anti-ACE (1:100, Proteintech, Germany GMBH) antibodies were used and incubated o/n at 4°C. Anti-rabbit Alexa Fluor™ -546-conjugated secondary antibody (1:500, ThermoFisher Scientific, CA, USA) was incubated at room temperature for 1 h. Antibodies were diluted in Antibody Diluent Reagent Solution (Life Techologies, ThermoFisher, CA, USA). Coverslips were mounted on glass slides with EverBrite™ Hardset Mounting Medium with DAPI (Biotium, Fremont, CA, USA) for subsequent observation at fluorescence microscope. The NIH ImageJ software was used to merge single channel images.

### Statistical Analysis

The results are expressed as the mean ± SD. To assess the significance between two series of data, ANOVA with Student’s t test was used. Statistical analysis was performed by GraphPad Prism 7.0 software and *P* < 0.05 was accepted as statistically significant.

## Results

### R1881 and Estradiol Promote TMPRSS2 Serine Protease Activation Without Affecting Total TMPRSS2 Expression

**Figure 1:**
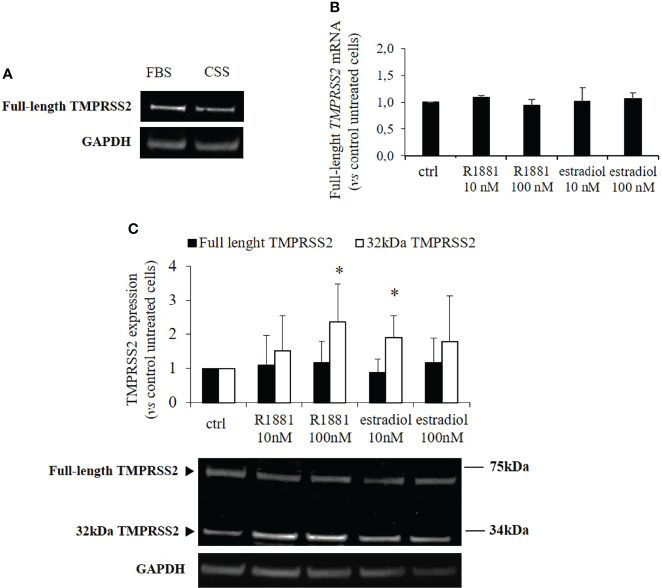
TMPRSS2 expression in response to stimulation with R1881 and estradiol. **(A)** Representative Western blot showing TMPRSS2 expression in Calu-3 cells cultured in complete medium or FBS-CSS medium for one week. **(B)** Analysis of TMPRRS2 mRNA expression in Calu-3 cells stimulated for 30 h with the indicated concentration of R1881 and estradiol in FBS-CSS medium. **(C)** Representative Western blot and densitometrical analysis of bands corresponding to the full-length form of TMPRSS2 and the 32kDa cleaved fragment containing the protease domain. Proteins were extracted from Calu-3 cells treated with the indicated concentration of R1881 and estradiol in FBS-CSS medium for 30 h. GAPDH was used as housekeeping gene for normalization. Experiments were performed in triplicate and results are expressed as mean ± SD. * = p < 0.05 vs control untreated cells.

Our clone of Calu-3 cells was previously tested for endogenous expression of TMPRSS2, ACE2, androgen receptor, estrogen receptor and aromatase (CY19A1). Calu-3 cells endogenously expressed all the mentioned genes with the exception of CY19A1 (data not shown). To evaluate whether sex-related hormones could differentially modulate TMPRSS2 expression and activity in Calu-3 cells, cells were first subjected to lipophilic hormones-deprivation for 1 week. Western blot analysis showed that under this culture condition TMPRSS2 expression was reduced compared with a not lipophilic hormones-deprived condition (**Figure 1A**). Cells were then stimulated with different concentrations of methyltrienolone (R1881) or estradiol to selectively test their effects on TMPRSS2 expression and activity. Preliminary experiments with different concentrations and incubation times were performed (**Supplementary Figures 1A-D**). No difference in mRNA and protein expression levels of full length TMPRSS2 was observed after R1881 and estradiol treatments (**Figures 1B, C**). Rather, TMPRSS2 auto proteolytic cleavage was promoted by both R1881 and estradiol since a significant increase in the intensity of the bands corresponding to the cleaved 32kDa TMPRSS2 fragment has been observed in cells exposed to 100 nM R1881 (+2.36 ± 1.13 fold-increase vs control untreated cells, p < 0.05) and 10 nM estradiol (+1.90 ± 0.64, fold-increase vs control untreated cells, p < 0.05), (**Figure 1C**). The effect exerted by R1881 was achieved at higher concentration compared to estradiol, although without a significative statistically difference.

To test a possible effect of R1881 and estradiol on TMPRSS2 intracellular localization, we performed immunofluorescence experiments. Our data showed that in basal condition TMPRSS2 was localized in the cytoplasm and plasma membrane, and no difference in its localization was observed after treatment with estradiol or R1881 (**Supplementary Figure 2**).

### R1881 and Estradiol Modulate Spike Protein Priming

**Figure 2:**
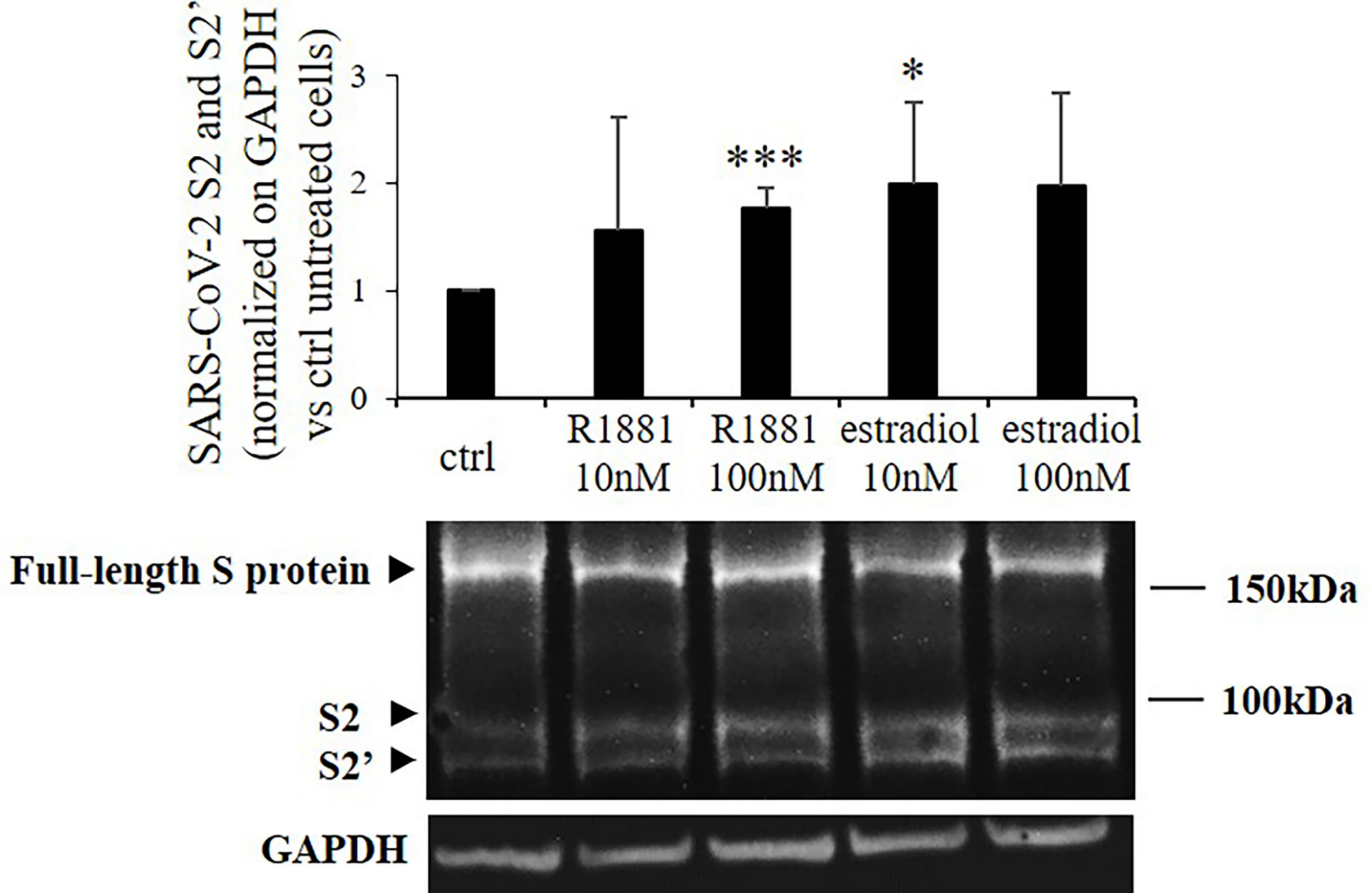
SARS-CoV-2 Spike cleavage in response to stimulation with R1881 and estradiol. Representative Western blot image and densitometrical analysis of bands corresponding to SARS-CoV-2 Spike cleavage products (S2 and S2’) in Calu-3 cells transfected with SARS-CoV-2 S plasmid and stimulated with the indicated concentration of R1881 and estradiol for 30 h in FBS-CSS medium. GAPDH was considered as housekeeping gene and was used for normalization. Experiments were replicated three times and results are expressed as mean ± SD. *, p < 0.05, ***, p < 0.001 vs control untreated cells.

Then we asked whether the increase in TMPRSS2 serine protease domain release mediated by sex-related hormones could result in enhanced SARS-CoV-2 Spike protein priming in Calu-3 cells. Indeed, TMPRSS2 activity is required for proteolytic processing of SARS-CoV-2 Spike at the S1/S2 and the S2’ sites in cell lines and subsequent SARS-CoV-2 infection of lung cells. To this purpose, Calu-3 cells kept in lipophilic hormone-deprived medium were transiently transfected with plasmid encoding SARS-CoV-2 Spike and then stimulated for 30 h with different concentrations of R1881 or estradiol. Western blot experiments on proteins from cell extracts showed significant increase in the expression levels of spike cleavage products after both 100 nM R1881 (+1.76 ± 0.18, p < 0.001 vs control untreated cells) and 10 nM estradiol (+1.99±,0.76 p < 0.05 vs control untreated cells) treatments (**Figure 2**). No statistically significative difference in the effects exerted by R1881 and estradiol was found.

### ACE2 Expression Is Not Affected by R1881 and Estradiol Cells Incubation

**Figure 3:**
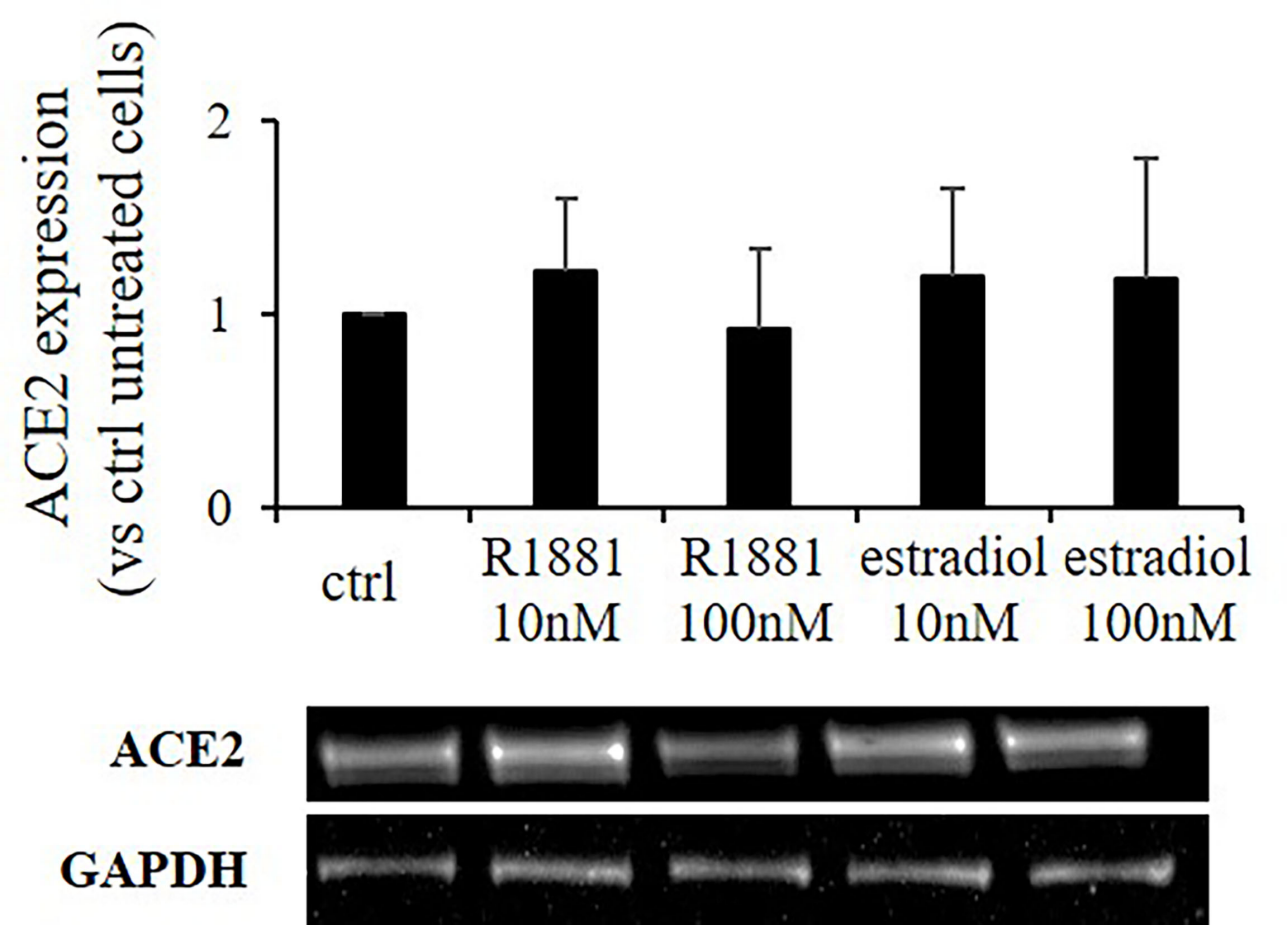
R1881 and estradiol do not influence ACE2 protein expression. Representative Western blot image and densitometrical analysis showing ACE2 protein expression in Calu-3 cells stimulated or not with the indicated concentration of R1881 and estradiol for 30 h in FBS-CSS medium. GAPDH was considered as housekeeping gene and was used for normalization. Experiments were replicated three times and results are expressed as mean ± SD.

SARS-CoV-2 Spike employs ACE2 as the entry receptor for host infection of target cells. We tested any possible effects of estradiol and R1881 on its expression. Western blot analysis was performed on Calu-3 cells deprived of lipophilic hormones for 1 week and subsequently stimulated with R1881 or estradiol at different doses. As reported in **Figure 3**, no significant differences in ACE2 expression were observed between hormones-stimulated cells and untreated control cells.

Immunofluorescence experiments showed that ACE2 was localized in the cytoplasm and plasma membrane in both basal condition and after estradiol or R1881 treatment (**Supplementary Figure 2**).

## Discussion

An unquestionable feature of the ongoing COVID-19 pandemic is the male bias towards the developing of a severe disease despite the same proportion of males and females infected with SARS-CoV-2. It is well known that immune responses to infection present with noteworthy differences between genders and are likely to be driving factors behind the significant sex-bias observed worldwide (27). Among the other biological factors involved, attention has been pointed on possible effects of sex-steroids on genetic regulation of TMPRSS2 and ACE2 expression, the two critical host cell mediators for the spread of SARS-CoV-2. In this regard, though several data documented an androgen-mediated upregulation of TMPRSS2 in the prostate cancer cells (19, 23), only a slight increase in TMPRSS2 transcript has been found in lung males compared to females (7). Other few and controversial observations have been made in lung cells (24, 26). However, a possible modulation of TMPRSS2 protein expression and activity by sex-related hormones in lung cells has not been investigated yet.

Therefore, in the present study we used lung Calu-3 cells to test androgen and estradiol effects on the modulation of TMPRSS2 and ACE2 expression levels, that in turn would influence SARS-CoV-2 host cell entry. As TMPRSS2 is an androgen responsive gene (7, 19, 20) but it is also regulated by estrogen (18) we first searched for possible changes in TMPRSS2 expression levels in Calu-3 cells stimulated with androgen or estrogen. At first, we could not find any substantial differences in TMPRSS2 mRNA levels, being this result in line with previous studies in lung cells (24) and human lung tissues showing a substantial equal expression patterns of the TMPRSS2 gene between males and females at different ages (7). Accordingly, we could not find differences in the expression levels of the full length 70kDa TMPRSS2 protein but, interestingly, we observed a statistically significant upregulation of TMPRSS2 cleavage upon both androgen and estrogen treatments. Similarly, treatment with mibolerone induced increase in the TMPRSS2 cleavage product in prostate cancer cells (14). Since TMPRSS2 cleavage is a consequence of its own catalytic activity (14, 15), our data suggested that both androgen and estrogen hormones could stimulate the protease function in Calu-3 cells, although a TMPRSS2 enzymatic activity assay could have provided a more definitive answer. Even if observed at a higher concentration, the effect exerted by R1881 seemed more pronounced if compared to estradiol. In Calu-3 cells, SARS-CoV-2-S was already shown to be a TMPRSS2 substrate (8). Here we demonstrated that, as a consequence of its functional activation promoted by androgen and estrogen hormones, TMPRSS2 significantly contributed to efficient proteolytic processing of Spike, generating the subunits S1 and S2. Given that our subclone of Calu-3 cells does not express aromatase we can assume that the increased TMPRSS2 cleavage observed in androgen treated cells was due to the action of androgen itself rather than its conversion into estradiol.

Up to date, several clinical trials are testing direct TMPRSS2 inhibitors as well as modulators of the sex-related hormones pathways indirectly acting on TMPRSS2 activity (28). Nevertheless, results from a meta-analysis conducted to evaluate the effect of androgen deprivation therapy (ADT) on COVID-19 in patients with prostate cancer failed to show a protective action against the risk of infection, hospital admission and mortality in these patients (29).

The S1 subunit of spike contains the ACE2 receptor binding domain, whereas the S2 subunit is anchored to the virus membrane and harbours the fusion machinery. We asked whether sex-related hormones could interfere with ACE2 expression in lung cells, as already seen in other tissues. Indeed, estrogen in atrial tissue has been shown modulate the local renin-angiotensin system *via* upregulation of ACE2 (30). Moreover, ACE2 has been identified as an AR-regulated target in certain types of cells in the lungs (24) and in prostate cancer cells, where both mRNA and protein expression of ACE2 were strongly upregulated by R1881 (25). However, we did not observe any difference in ACE2 expression levels in Calu-3 cells stimulated with estradiol or R188. Similarly, no difference was found in human lung ACE2 transcripts between males and females (7, 31). In our case, it is possible to speculate that the absence of considerable changes in ACE2 expression levels observed upon stimulation with hormones might be due to the abundant levels of this receptor in Calu-3 cells compared to other cell lines (25, 32). TMPRSS2 and HAT proteases can mediate ACE2 proteolytic cleavage in order to augment SARS-S-driven entry (33). Specifically, the 13-kDa fragment of ACE2 has been detected in HEK293 cells transiently transfected with TMPRSS2 or HAT, being this efficiency of ACE2 cleavage dependent on the proteases expression level (33). Here, we also searched for the production of cleaved fragments of ACE2, but none of them were detectable nor under basal condition neither after treatment with hormones. Thus, it is possible to hypothesize that TMPRSS2 levels in Calu-3 cells were not sufficient enough to promote the C-terminal processing of ACE2.

Overall, our data are in agreement with the lack of difference in the percentage of males and females infected with SARS-CoV-2 globally reported (27). However, why males are exposed to higher odds of both intensive therapy unit (ITU) admission and death compared to females is a still an unsolved question and the mechanism underlying the gender disparity in COVID-19 outcomes is expected to be multifactorial. Thus, understanding of the factors that modulate susceptibility to SARS-CoV-2 remains crucial for controlling disease transmission and health consequences.

## Data Availability Statement

The original contributions presented in the study are included in the article/**Supplementary Material**. Further inquiries can be directed to the corresponding author.

## Author Contributions

Conceptualization, EP and GioM; investigation, DT, GiuM, GDM, RC, FM, EE, AB; writing—original draft preparation, DT; writing-review and editing, EP and GioM; supervision, MA. All authors contributed to manuscript revision, read, and approved the submitted version.

## Funding

The work was supported by Ricerca Corrente Funds from the Italian Ministry of Health to Fondazione IRCCS Ca’ Granda Ospedale Maggiore Policlinico, Milan.

## Conflict of Interest

The authors declare that the research was conducted in the absence of any commercial or financial relationships that could be construed as a potential conflict of interest.

## Publisher’s Note

All claims expressed in this article are solely those of the authors and do not necessarily represent those of their affiliated organizations, or those of the publisher, the editors and the reviewers. Any product that may be evaluated in this article, or claim that may be made by its manufacturer, is not guaranteed or endorsed by the publisher.
