# Supplementary material for: TMPRSS2 Expression and Activity Modulation by Sex-Related Hormones in Lung Calu-3 Cells: Impact on Gender-Specific SARS-CoV-2 Infection
Source: Front Endocrinol (Lausanne). 2022 May 31;13:862789. doi: 10.3389/fendo.2022.862789 (PMC9193185; doi:10.3389/fendo.2022.862789)
Supplement: Supplementary file 1 [file Presentation_1.pptx]

## Slide 1
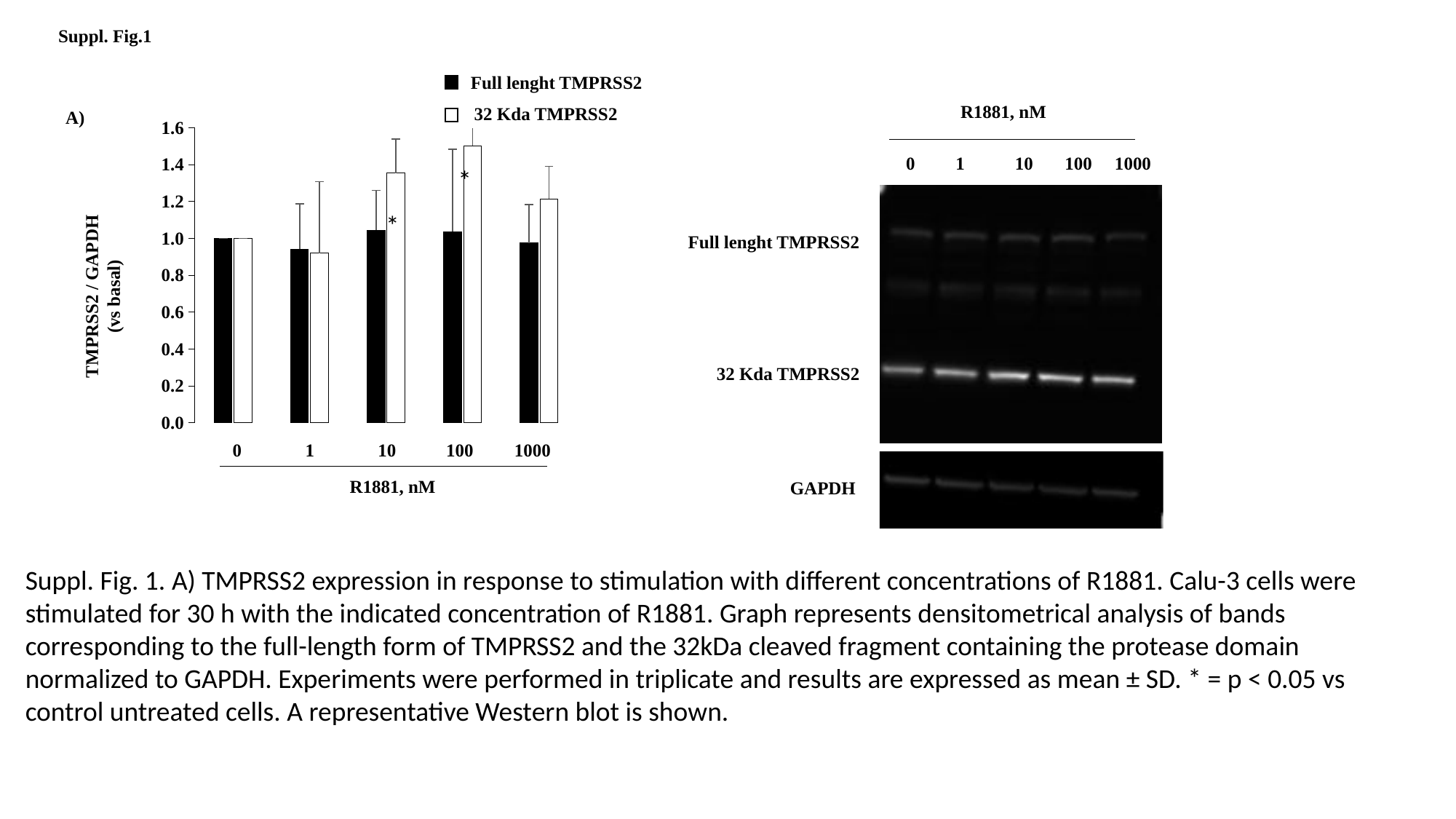

Suppl. Fig.1
Full lenght TMPRSS2
R1881, nM
32 Kda TMPRSS2
A)
### Chart
| Category | | |
|---|---|---|0 1 10 100 1000
*
*
Full lenght TMPRSS2
TMPRSS2 / GAPDH
(vs basal)
32 Kda TMPRSS2
0 1 10 100 1000
R1881, nM
GAPDH
Suppl. Fig. 1. A) TMPRSS2 expression in response to stimulation with different concentrations of R1881. Calu-3 cells were stimulated for 30 h with the indicated concentration of R1881. Graph represents densitometrical analysis of bands corresponding to the full-length form of TMPRSS2 and the 32kDa cleaved fragment containing the protease domain normalized to GAPDH. Experiments were performed in triplicate and results are expressed as mean ± SD. * = p < 0.05 vs control untreated cells. A representative Western blot is shown.

## Slide 2
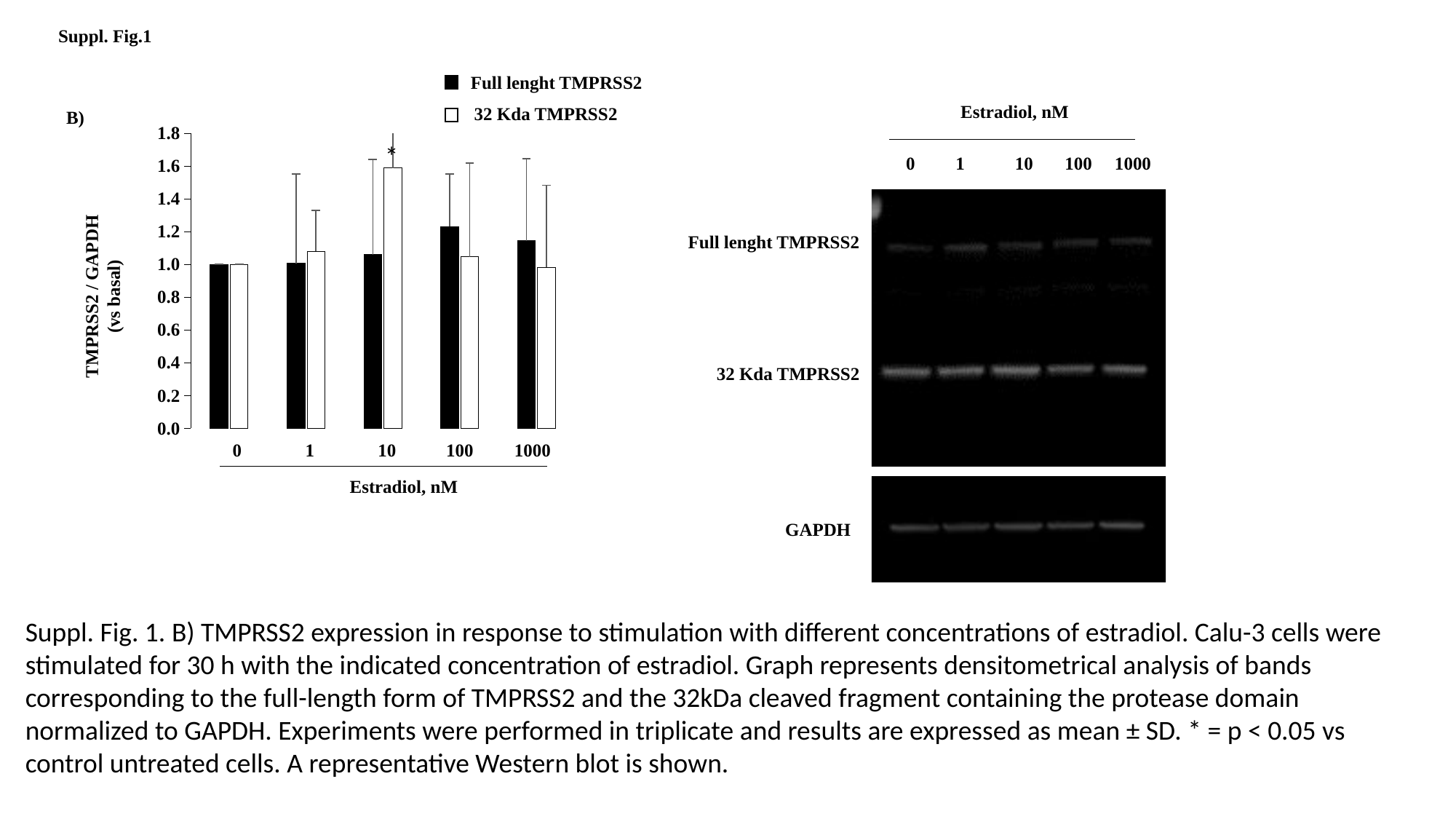

Suppl. Fig.1
Full lenght TMPRSS2
Estradiol, nM
32 Kda TMPRSS2
B)
### Chart
| Category | | |
|---|---|---|*
0 1 10 100 1000
Full lenght TMPRSS2
TMPRSS2 / GAPDH
(vs basal)
32 Kda TMPRSS2
0 1 10 100 1000
Estradiol, nM
GAPDH
Suppl. Fig. 1. B) TMPRSS2 expression in response to stimulation with different concentrations of estradiol. Calu-3 cells were stimulated for 30 h with the indicated concentration of estradiol. Graph represents densitometrical analysis of bands corresponding to the full-length form of TMPRSS2 and the 32kDa cleaved fragment containing the protease domain normalized to GAPDH. Experiments were performed in triplicate and results are expressed as mean ± SD. * = p < 0.05 vs control untreated cells. A representative Western blot is shown.

## Slide 3
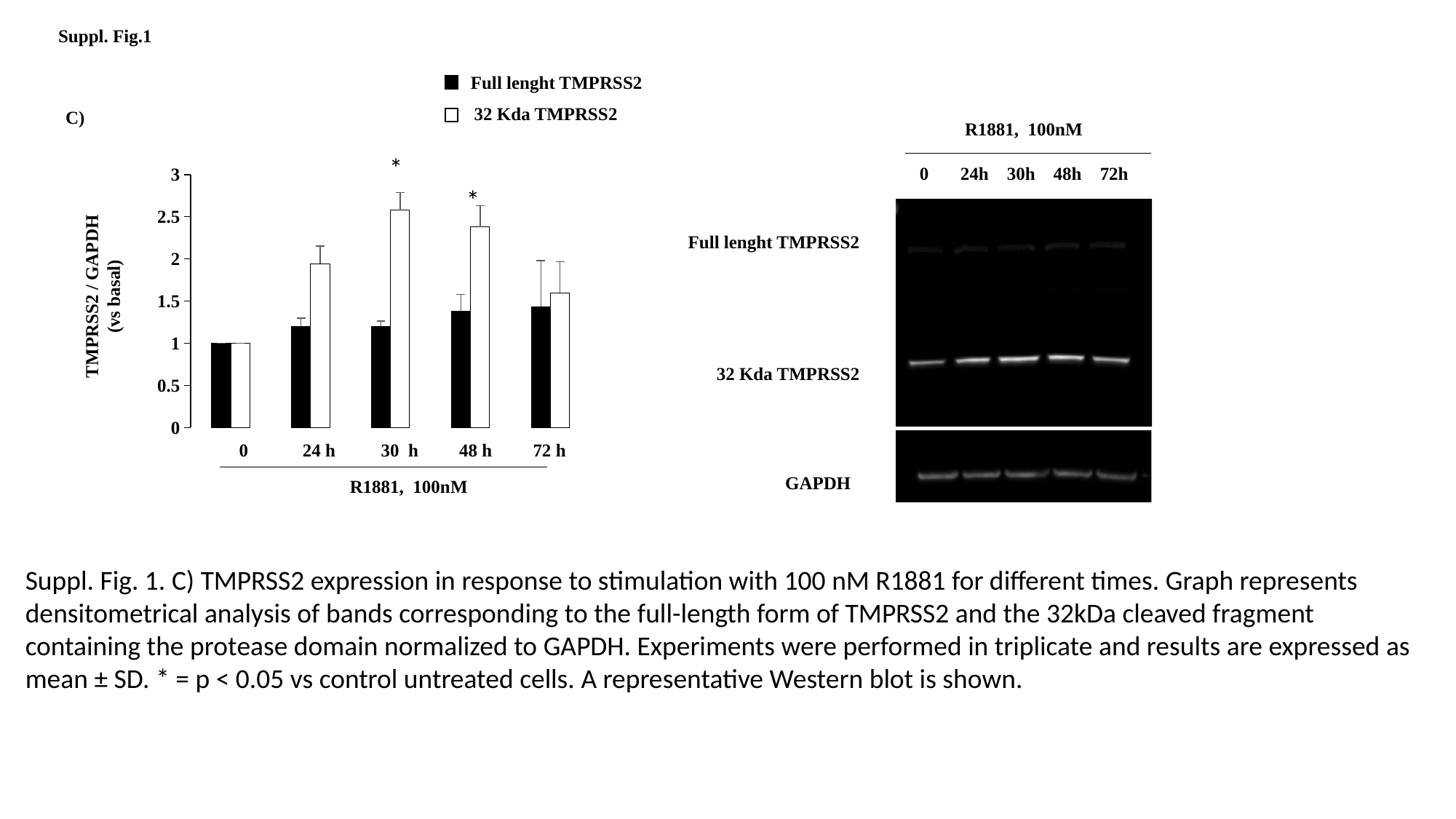

Suppl. Fig.1
Full lenght TMPRSS2
32 Kda TMPRSS2
C)
R1881, 100nM
*
0 24h 30h 48h 72h
### Chart
| Category | | |
|---|---|---|*
Full lenght TMPRSS2
TMPRSS2 / GAPDH
(vs basal)
32 Kda TMPRSS2
0 24 h 30 h 48 h 72 h
GAPDH
R1881, 100nM
Suppl. Fig. 1. C) TMPRSS2 expression in response to stimulation with 100 nM R1881 for different times. Graph represents densitometrical analysis of bands corresponding to the full-length form of TMPRSS2 and the 32kDa cleaved fragment containing the protease domain normalized to GAPDH. Experiments were performed in triplicate and results are expressed as mean ± SD. * = p < 0.05 vs control untreated cells. A representative Western blot is shown.

## Slide 4
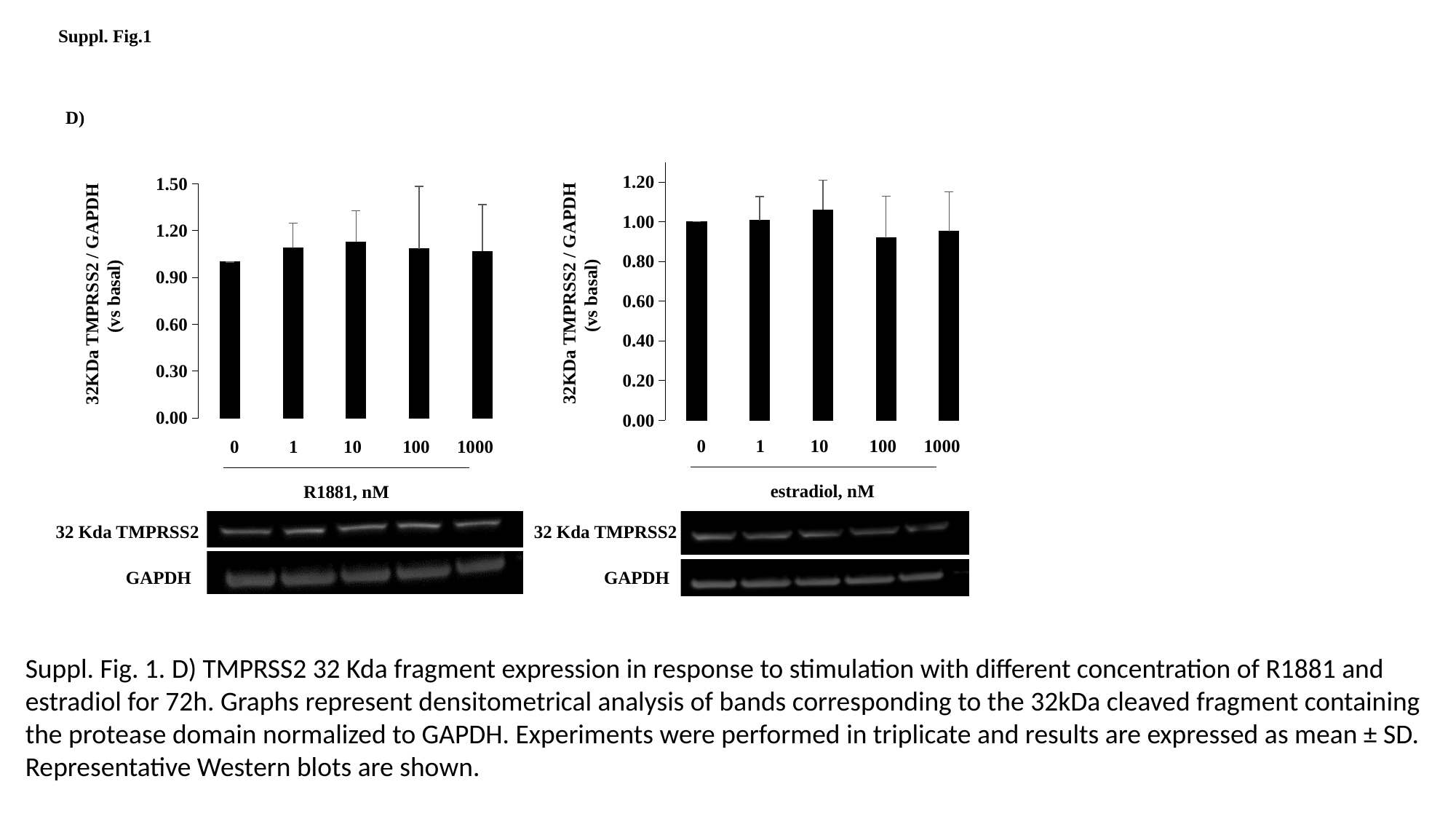

Suppl. Fig.1
D)
### Chart
| Category | |
|---|---|
| Bas | 1.0 |
| Estr 1nM | 1.006909181649836 |
| Estr 10nM | 1.06 |
| Estr 100nM | 0.92 |
| Estr 1uM | 0.9520959821633345 |
### Chart
| Category | |
|---|---|
| Bas | 1.0 |
| R1881 1nM | 1.089025968652731 |
| R1881 10nM | 1.1284589527472835 |
| R1881 100nM | 1.0838693145928593 |
| R1881 1uM | 1.0684714801110617 | 32KDa TMPRSS2 / GAPDH
(vs basal)
 32KDa TMPRSS2 / GAPDH
(vs basal)
0 1 10 100 1000
0 1 10 100 1000
estradiol, nM
R1881, nM
32 Kda TMPRSS2
32 Kda TMPRSS2
GAPDH
GAPDH
Suppl. Fig. 1. D) TMPRSS2 32 Kda fragment expression in response to stimulation with different concentration of R1881 and estradiol for 72h. Graphs represent densitometrical analysis of bands corresponding to the 32kDa cleaved fragment containing the protease domain normalized to GAPDH. Experiments were performed in triplicate and results are expressed as mean ± SD. Representative Western blots are shown.
