# Supplementary material for: TMPRSS2 Expression and Activity Modulation by Sex-Related Hormones in Lung Calu-3 Cells: Impact on Gender-Specific SARS-CoV-2 Infection
Source: Front Endocrinol (Lausanne). 2022 May 31;13:862789. doi: 10.3389/fendo.2022.862789 (PMC9193185; doi:10.3389/fendo.2022.862789)
Supplement: Supplementary file 2 [file Presentation_2.pptx]

## Slide 1
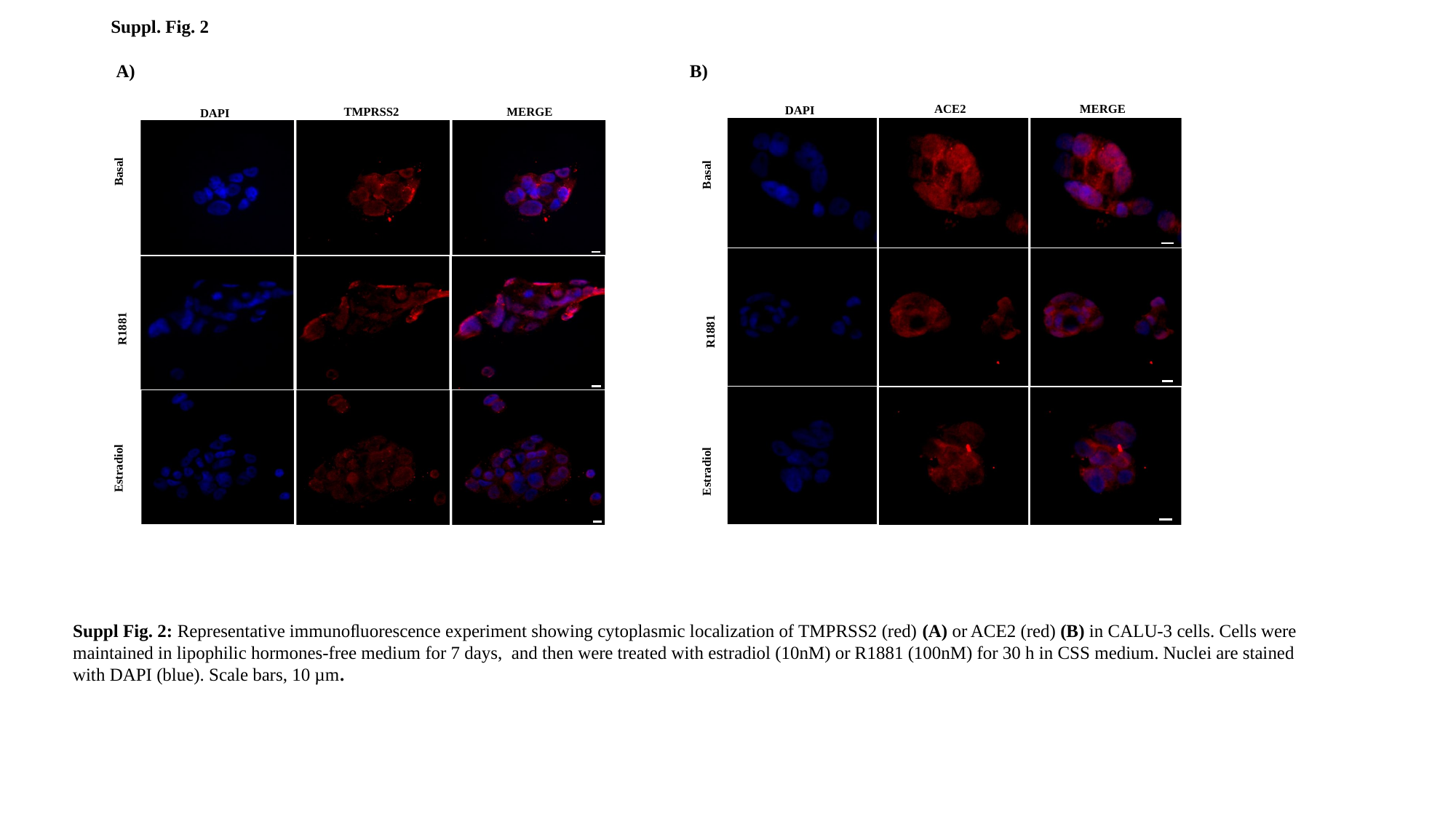

Suppl. Fig. 2
A)
B)
Basal
R1881
MERGE
TMPRSS2
DAPI
Estradiol
MERGE
ACE2
DAPI
10μM
10μM
Basal
R1881
Estradiol
Suppl Fig. 2: Representative immunoﬂuorescence experiment showing cytoplasmic localization of TMPRSS2 (red) (A) or ACE2 (red) (B) in CALU-3 cells. Cells were maintained in lipophilic hormones-free medium for 7 days, and then were treated with estradiol (10nM) or R1881 (100nM) for 30 h in CSS medium. Nuclei are stained with DAPI (blue). Scale bars, 10 µm.
